# Do maternal BMI and gestational weight gain equally affect the risk of infant hypoxic and traumatic events?

**DOI:** 10.1371/journal.pone.0308441

**Published:** 2024-08-06

**Authors:** Giuseppe Chiossi, Riccardo Cuoghi Costantini, Daniela Menichini, Anna Luna Tramontano, Marialaura Diamanti, Fabio Facchinetti, Roberto D’Amico

**Affiliations:** 1 Department of Medical and Surgical Sciences for Mother, Division of Obstetrics, Child and Adult, University of Modena and Reggio Emilia, Modena, Italy; 2 Department of Diagnostic and Clinical Medicine and Public Health, Statistics Unit, University of Modena and Reggio Emilia, Modena, Italy; University of Tennessee Health Science Center, UNITED STATES OF AMERICA

## Abstract

**Background:**

Small (SGA) and large (LGA) for gestational age infants have higher risks of infant morbidity when compared to those who are appropriate for gestational age (AGA). Increasing pre-pregnancy maternal BMI and gestational weight gain (GWG) are associated with higher risks of LGA and lower risks of SGA infants; however, their direct effects on infant morbidity are unknown. Therefore, we intended to 1) assess how maternal pre-pregnancy BMI, GWG, and birthweight (categorized as SGA, AGA or LGA) affect infant morbidity and 2) estimate at entry of care the risk of infant morbidity according to pre-pregnancy BMI and possible GWG.

**Methods:**

we used Consortium on Safe Labor data, a retrospective observational cohort study collecting pregnancy and birth data from 2002 to 2008 in 12 US centers. The association between maternal BMI, GWG and infant morbidity was estimated in singleton gestations delivering ≥ 37 weeks using binomial logistic regression. Hypoxic composite neonatal morbidity was defined as any the following: stillbirth, neonatal death, resuscitation at birth, NICU admission, intracranial hemorrhage, PVH grade III and IV, neonatal seizures, NEC, meconium aspiration, CPAP or mechanical ventilation, RDS, and sepsis. Traumatic composite neonatal morbidity included shoulder dystocia or birth injuries.

**Results:**

In this study of 110,594 mother-infant dyads, a total of 8,369 (7.6%) infants experienced hypoxic, while 2,134 (1.9%) developed traumatic morbidity. The risk of hypoxic morbidity among SGA, AGA and LGA infants increased when mothers were overweight (aOR 1.26 [95%CI 1.18–1.34]) or obese (class 1: aOR 1.3 [1.2–1.4]; class 2: aOR 1.7 [1.5–1.9]; class 3: aOR 1.8 [1.6–2]) as opposed to normal weight, and when GWG exceeded (aOR 1.08 [1.02–1.014]) rather than remained within recommendations. The risk of traumatic morbidity increased with maternal obesity (class 1: aOR 1.3 [1.1–1.5]), whilst it dropped with GWG below recommendations (aOR 0.7 [0.6–0.8]). The risk of hypoxic events estimated at entry of care increased with maternal overweight (aOR 1.27 [1.19–1.35]) or obesity (class 1: aOR 1.4 [1.2–1.5]; class 2: aOR 1.7 [1.5–1.9]; class 3: aOR 1.8 [1.6–2.1]), and with possible GWG above (aOR 1.09 [1.03–1.015]) recommendations. The risk of traumatic morbidity increased with overweight (aOR 1.1 [1–1.3]) or obesity (class 1: aOR 1.4 [1.2–1.6]; class 2: aOR 1.3 [1–1.6]), with possible GWG above (aOR 1.2 [1–1.3]), as opposed to below recommendations (aOR 0.7 [0.6–0.8]).

**Conclusions:**

While maternal pre-pregnancy BMI and GWG equally affected traumatic morbidity, the former had a greater impact on hypoxic complications. Therefore, weight control prior to pregnancy is at least as effective as avoiding excessive gestational weight gain to prevent neonatal morbidity.

## Introduction

Pre-pregnancy body mass index (BMI; calculated as weight in kilograms divided by height in meters squared) and gestational weight gain (GWG) affect the risk of having small (i.e. birthweight < 10^th^ percentile, SGA) and large (i.e. birthweight > 90^th^ percentile, LGA) for gestational age infants, as the prevalence of SGA drops with higher maternal BMI and pregnancy weight gain, while LGA’s increases [1,2]. Although most SGA and LGA have uncomplicated deliveries and require standard neonatal care, they are often considered as the results of pregnancy complications [3,4] having higher morbidity and mortality than appropriate for gestational age infants (AGA; birthweight at 10-90^th^ percentile) [5]. Specifically, SGA are at increased risk for stillbirth, seizures, sepsis, intraventricular hemorrhage (IVH), necrotizing enterocolitis (NEC), hypoxic ischemic encephalopathy and neonatal mortality, when compared to AGA [5,6]; LGA instead are at higher risk of stillbirth, traumatic delivery, brachial plexus palsy and neonatal mortality [5,7]. Therefore, the US National Academy of Medicine identified for each maternal BMI category the GWG that minimizes their risk of having SGA and LGA infants [8], even if the contribution of pre-pregnancy BMI and GWG to infant morbidity is still a matter of debate in these birthweight categories.

By contrast, investigating the direct effects of maternal pre-pregnancy BMI and GWG on neonatal health would provide more useful information than the one provided by intermediate outcomes such as birthweight [9]. This seems particularly relevant as the prevalence of obesity, which is a risk factor for LGA, has reached 39.7% among women of reproductive age in the United States [10], and the association of GWG with infants’ adverse outcomes is rarely stratified by maternal BMI [9,11–13] or by the severity of obesity [9,11]. Furthermore, maternal conditions impacting birthweight, such as diabetes mellitus, hypertension and obesity, rather than birthweight itself, may account for SGA and LGA’s adverse outcomes [14–16]. As prenatal care is focused on promoting healthy births, investigating the effects of maternal BMI and GWG on infant morbidity could be used not only to predict adverse outcomes, but also to counsel pregnant women, and to enable obstetric providers to identify at risk pregnancies.

Therefore, using a large retrospective cohort of pregnant women, this study intends to: 1) assess how pre-pregnancy BMI, GWG, and birthweight affect infant morbidity; 2) estimate at entry of care the risk of infant morbidity and mortality according to pre-pregnancy BMI and possible GWG.

## Materials and methods

We used data from the Consortium on Safe Labor, a multicenter retrospective observational study that abstracted detailed labor and delivery information from electronic medical records in 12 US clinical centers (with 19 hospitals), from 2002 to 2008, available from the National Institute of Child and Human Development Data and Specimen Hub [NICHD DASH, 17]. The original study was approved by the institutional review boards (IRB) of all participating institutions; a detailed description of its design was provided elsewhere [18]. As the survey represented a retrospective analysis of medical records, the IRBs of all participating institutions waived the requirement for informed consent; furthermore, data were fully anonymized before they were accessed for the primary analysis. As we conducted a secondary analysis of previously published fully anonymized data, the study protocol was exempted from IRB approval at our institution.

In brief, maternal demographic characteristics, medical, reproductive, and prenatal history, delivery outcomes, neonatal characteristics, and postpartum complications were extracted from hospital electronic medical records and discharge summaries. International Classification of Diseases, Ninth Revision (ICD-9) codes were abstracted for both mothers and infants.

BMI categories were determined by World Health Organization criteria (underweight <18.5 kg/m^2^, normal 18.5–24.9 kg/m^2^, overweight 25.0–29.9 kg/m^2^, obese Class 1 30.0–34.9 kg/m^2^, obese Class 2 35.0–39.9 kg/m^2^, and obese Class 3 ≥ 40 kg/m^2^) [19]. GWG was categorized as above, within or below the IOM recommendations according to women’s pre-pregnancy BMI: the recommended amount is 12.5–18 kg for underweight women, 11.5–16 kg for those entering pregnancy with normal BMI, 7–11.5 kg for overweight women, and 5–9 kg for obese mothers-to-be [8]. Infants were categorized as AGA, SGA and LGA according to their birthweight as described by Alexander et al [20].

Maternal socio-demographic characteristics (maternal BMI category, pregnancy weight gain, age, race/ethnicity, marital status, health insurance, education, smoking, alcohol, and drug use), maternal comorbidities (asthma, depression, anemia, hypertensive disorders, and diabetes mellitus), obstetric features (parity, gestational age at delivery, type of labor, mode of delivery) and newborn data (newborns’ sex and weight) were described.

Singleton pregnancies with gestational age at delivery ≥ 37 weeks’ gestation were analyzed. Infant morbidity was measured as composite outcomes related to hypoxic and traumatic neonatal events, according to the work of Chauhan et al [5]. Hypoxic composite neonatal morbidity was defined by the occurrence of at least one of the following events: stillbirth, neonatal death, resuscitation at birth, NICU admission, intracranial hemorrhage, periventricular hemorrhage (PVH) grade III and IV, neonatal seizures, NEC, meconium aspiration, non-invasive (CPAP) or invasive (mechanical ventilation) respiratory support, respiratory distress syndrome (RDS), and neonatal sepsis. Traumatic composite neonatal morbidity was defined by the occurrence of shoulder dystocia or neonatal birth injury.

Descriptive statistics were used for all study variables. Categorical variables were presented as absolute and percentage frequencies, continuous variables were summarized as mean ± SD. Chi square test or Fisher exact test were used to test associations between outcomes and birthweight category.

We used binomial logistic regression models to investigate the risk of hypoxic and traumatic composite neonatal morbidities according to maternal pre-pregnancy BMI, GWG, and birthweight, accounting for confounders such as, maternal age, ethnicity, marital status, parity, smoking, alcohol or drug use, and sex of the infant. We only considered covariates that were recorded in > 90% of the mother-infant dyads. Only statistically significant covariates were included in the final model. A level of significance of p < 0.05 was considered. The possible interaction between pre-pregnancy BMI and GWG was also investigated. We decided against adjusting for possible mediators between maternal BMI, GWG and infant outcomes, such as maternal morbidities, labor complications, or mode of delivery [3,21]. We also used binomial logistic regression models to estimate at entry of care the risk of hypoxic and traumatic composite neonatal morbidities according to maternal pre-pregnancy BMI, possible GWGs, and the previously mentioned confounders. The models did not include birthweight as it only becomes available at the time of delivery. Calibration and discrimination of the models were measured by using the area under the curve (AUC) and the Hosmer-Lemeshow (HL) test. To determine if pre-pregnancy BMI and GWG equally affected infant morbidity, we compared the AUC of the model assessing the association between BMI and infant morbidity (including all the covariates but maternal GWG) with the AUC of the model assessing the association between GWG and infant morbidity (including all the covariates but BMI) [22].

This study followed the Strengthening the Reporting of Observational Studies in Epidemiology (STROBE) reporting guideline for cohort studies [23]. All statistical analyses were performed using STATA 15 (College Station, TX).

## Results

There were a total of 228,668 deliveries (87% of which occurred during 2005–2007) in the database. For the current study, births were excluded based on the following criteria: 1) deliveries other than the 1^st^ (for those who had multiple pregnancies during the study period, only data on the 1^st^ delivery were included in the analysis to avoid intra-person correlation): 19,773; 2) multiple pregnancies: 5,191; 3) congenital anomalies or aneuploidies: 14,290; 4) gestational age at delivery < 37 weeks’ gestation: 20,143; 4) unknown maternal pre-pregnancy BMI: 53,972; 5) unknown maternal GWG: 3,450, 6) GWG greater than the mean maternal weight gain + 3SD or GWG lower than the mean maternal weight gain– 3SD: 75, this criterium was adopted to remove potential errors in database entry. It is worth noting that some deliveries met more than one exclusion criteria. The 110,594 remaining mother-infant dyads represented our study sample.

Demographic data and pregnancy characteristics are presented in Table 1A and 1B.

**Table 1:** A: Socio-demographic characteristics of the study population. B: Medical, obstetrics and delivery characteristics of the study population.

**Characteristics**		**Missing data** (% of the 110594 women enrolled)
**Maternal age** (n = 110524)	27.61 ± 6.09	70 (*<*0.01%)
**Ethnicity** (n = 110594)		0 (0%)
White	58647 (53.03%)	
Black	20194 (18.26%)	
Hispanic	21489 (19.43%)	
Other	10264 (9.28%)	
**Marital status** (n = 108708)		1886 (1.71%)
Unmarried	38235 (35.17%)	
Married	70473 (64.83%)	
**Health Insurance** (n = 93904)		16690 (15.09%)
Private	59222 (63.07%)	
Public/Self-pay	34682 (36.93%)	
**Education** (n = 52652)		58942 (53.3%)
Highschool or less	25102 (47.7%)	
Above Highschool	27550 (52.3%)	
**Smoking** (n = 110594)	6776 (6.13%)	0 (0%)
**Alcohol** (n = 110594)	1979 (1.79%)	0(0%)
**Drugs** (n = 100007)	1642 (1.64%)	10587 (9.57%)
**BMI category** (n = 110594)		0 (0%)
Underweight	5995 (5.42%)	
Normal weight	60666 (54.85%)	
Overweight	24609 (22.25%)	
Obesity class 1	11180 (10.11%)	
Obesity class 2	4885 (4.42%)	
Obesity class 3	3259 (2.95%)	
**Characteristics**		**Missing data** (% of the 110594 women enrolled)
**Asthma** (n = 104816)	6622 (6.32%)	5778 (5.22%)
**Depression** (n = 110594)	4246 (3.84%)	0 (0%)
**Anemia** (n = 94405)	6925 (7.33%)	16189 (14.64%)
**Chronic Hypertension** ± **Superimposed Preeclampsia** (n = 100095)	2468 (2.47%)	10499 (9.49%)
**Gestational Hypertension, Preeclampsia, HELLP syndrome** (n = 94266)	4479 (4.75%)	16328 (14.76%)
**Pregestational diabetes** (n = 104816)	1747 (1.67%)	5778 (5.22%)
**Gestational diabetes** (n = 89596)		20998 (18.99%)
Class A1	1358 (1.52%)	
Class A2	995 (1.11%)	
**Nulliparous** (n = 110594)	48153 (43.54%)	0 (0%)
**Gestational age at delivery (weeks)** (n = 110594)		0 (0%)
37	11437 (10.34%)	
38	24895 (22.51%)	
39	39917 (36.09%)	
40	25288 (22.87%)	
41	8216 (7.43%)	
42	841 (0.76%)	
**Gestational weight gain**		0 (0%)
Below IOM recommendations	20437 (18.48%)	
Within IOM recommendations	35179 (31.81%)	
Above IOM recommendations	54978 (49.71%)	
**Type of labor** (n = 110594)		0 (0%)
No labor	9494 (8.58%)	
Spontaneous labor	57.672 (52.15%)	
Induced labor	43.428 (39.27%)	
**Mode of delivery** (n = 110594)		0 (0%)
Vaginal	75319 (68.10%)	
Operative vaginal	6774 (6.13%)	
Cesarean	28501 (25.77%)	
**Sex of the infant** (n = 110594)		0 (0%)
Female	54572 (49.34%)	
Male	55928 (50.57%)	
Undetermined	94 (0.08)	
**Birthweight** (n = 110016)		578 (0.52%)
Female infants	3306 ± 433	
Male infants	3434 ± 450	

Data are displayed as mean ± SD, or n (%).

The average maternal age was 27.6 years, most mothers-to-be were white, married, and had private insurance; approximately 6% were smokers while less than 2% were respectively drugs or alcohol users. More than half of the women had normal BMI, followed by overweight and obesity, while only 5% were underweight. While approximately one-third of women (31.81%) gained weight within the IOM recommendations, the remaining two-thirds were outside, 49.71% being above, while 18.48% below. All deliveries were term, with approximately one third at 39 weeks.

As displayed in Table 2, hypoxic events were more frequent than traumatic complications, being respectively 7.57% and 1.93% the composite neonatal morbidity rates.

**Table 2:** Hypoxic and traumatic neonatal morbidity.

	SGA (n = 11289)	AGA (n = 88351)	LGA (n = 10954)	Total (n = 110594)	P
**Hypoxic composite neonatal morbidity**	1162 (10.29%)	6102 (6.91%)	1105 (10.09%)	8369 (7.57%)	< 0.01^#^
**Traumatic composite neonatal morbidity**	98 (0.86%)	1446 (1.64%)	590 (5.39%)	2134 (1.93%)	< 0.01^#^
**Details of the composite outcome**					
** Shoulder dystocia**	26 (0.23%)	790 (0.79%)	507 (4.63%)	1323 (1.2%)	< 0.01^#^
** Neonatal birth injury**	72 (0.64%)	684 (0.77%)	103 (0.94%)	859 (0.78%)	0.04^#^
** Stillbirth**	43 (0.38%)	136 (0.15%)	25 (0.23%)	204 (0.18%)	< 0.01^#^
** Neonatal death**	0 (0%)	3 (<0.01%)	1 (0.01%)	4 (<0.01%)	0.3*
** Resuscitation**	378 (3.35%)	2204 (2.49%)	331 (3.02%)	2913 (2.63%)	< 0.01^#^
** NICU Admission**	907 (8.03%)	4485 (5.08%)	888 (8.11%)	6280 (5.68%)	< 0.01^#^
** Mechanical ventilation**	99 (0.88%)	319 (0.36%)	45 (0.41%)	463 (0.42%)	< 0.01^#^
** CPAP**	67 (0.60%)	288 (0.33%)	46 (0.42%)	401 (0.36%)	< 0.01^#^
** Intracranial hemorrhage**	15 (0.13%)	46 (0.05%)	3 (0.03%)	64 (0.06%)	< 0.01^#^
** Periventricular hemorrhage grade III and IV**	0 (0%)	4 (<0.01%)	0 (0%)	4 (<0.01%)	< 0.01*
** RDS**	101 (0.89%)	416 (0.47%)	61 (0.56%)	578 (0.52%)	< 0.01^#^
** Neonatal seizures**	14 (0.12%)	57 (0.06%)	6 (0.05%)	77 (0.07%)	< 0.01^#^
** NEC**	5 (0.04%)	10 (0.01%)	2 (0.02%)	17 (0.02%)	< 0.01*
** Meconium aspiration**	37 (0.33%)	152 (0.17%)	35 (0.32%)	214 (0.19%)	< 0.01^#^
** Neonatal sepsis**	130 (1.16%)	629 (0.71%)	137 (1.26%)	896 (0.81%)	< 0.01^#^

Data are displayed as n (%)

^#^Chi square test

*Fisher exact test.

Outcomes differed according to birthweight categories: hypoxic composite neonatal morbidity reached 10.29% among SGA, 6.91% among AGA, and 10.09% among LGA (p < 0.01), while traumatic composite neonatal morbidity was detected in 0.86% of SGA, 1.64% of AGA and 5.39% of LGA (p < 0.01). LGA more frequently experienced shoulder dystocia (p < 0.01) and birth injuries (p = 0.04). SGA were more susceptible to develop stillbirth, intracranial hemorrhage, RDS, seizures, as well as NEC (p < 0.01), and they more frequently received invasive and non-invasive respiratory support (p < 0.01). AGA infants were less likely to undergo neonatal resuscitation and NICU admission when compared to SGA and LGA (p < 0.01), and they were less commonly diagnosed with meconium aspiration or neonatal sepsis (p < 0.01). Only 4 neonatal deaths were observed: 3 among AGA and 1 among LGA. Four AGA infants were diagnosed with grade III or IV PVH.

Table 3A shows that maternal pre-pregnancy BMI had a greater impact on hypoxic morbidity than GWG: the crude rates of the composite outcome almost doubled from 6.59% among underweight women to 11.78% among obese class 3 mothers, while they only varied from 6.79% to 8.3% when GWG did not reach or exceeded IOM recommendations. On the contrary, the crude rates of composite traumatic neonatal morbidity were less susceptible to changes in maternal pre-pregnancy BMI (ranging from 1.72% among underweight women to 2.44% among obese class 2 mothers), while they progressively increased with GWG (from 1.26% in case of GWG below IOM recommendations to 2.28% if GWG exceeded recommendations) (Table 3B).

**Table 3:** A: Hypoxic neonatal morbidity according to maternal pre-pregnancy BMI and GWG. B: Traumatic neonatal morbidity according to maternal pre-pregnancy BMI and GWG.

Pre-pregnancy	Subjects/	GWG according to IOM recommendations			Total
BMI	Events	Below	Within	Above	
Underweight	N	1798	2518	1679	5995
	N Composite	105	168	122	395
	% Composite	5.84	6.67	7.27	**6.59**
Normal weight	N	12428	23448	24790	60666
	N Composite	778	1499	1797	4074
	% Composite	6.26	6.39	7.25	**6.72**
Overweight	N	2578	5157	16874	24609
	N Composite	182	374	1469	2025
	% Composite	7.06	7.25	8.71	**8.23**
Obesity class 1	N	1542	2214	7424	11180
	N Composite	109	184	693	986
	% Composite	7.07	8.31	9.33	**8.82**
Obesity class 2	N	1125	1131	2629	4885
	N Composite	110	102	293	505
	% Composite	9.78	9.02	11.14	**10.34**
Obesity class 3	N	966	711	1582	3259
	N Composite	104	92	188	384
	% Composite	10.77	12.94	11.88	**11.78**
**Total**	N	20437	35179	54978	110594
	N Composite	1388	2419	4562	8369
	% Composite	**6.79**	**6.88**	**8.30**	**7.57**
**Pre-pregnancy**	**Subjects/**	**GWG according to IOM recommendations**			**Total**
**BMI**	**Events**	**Below**	**Respected**	**Above**	
Underweight	N	1798	2518	1679	5995
	N Composite	19	44	40	103
	% Composite	1.06	1.75	2.38	**1.72**
Normal weight	N	12428	23448	24790	60666
	N Composite	144	382	525	1051
	% Composite	1.16	1.63	2.12	1.73
Overweight	N	2578	5157	16874	24609
	N Composite	28	103	384	515
	% Composite	1.09	2.00	2.28	**2.09**
Obesity class 1	N	1542	2214	7424	11180
	N Composite	25	57	202	284
	% Composite	1.62	2.57	2.72	**2.54**
Obesity class 2	N	1125	1131	2629	4885
	N Composite	28	26	65	119
	% Compl	2.49	2.30	2.47	**2.44**
Obesity class 3	N	966	711	1582	3259
	N Composite	14	13	35	62
	% Composite	1.45	1.83	2.21	**1.90**
**Total**	N	20437	35179	54978	110594
	N Composite	258	625	1251	2134
	% Composite	**1.26**	**1.78**	**2.28**	**1.93**

N: Number of pregnant women, N Composite: Number of pregnant women developing the composite outcome, % Composite: Percentage of pregnant women developing the composite outcome.

On multivariable analysis (Table 4), the risk of developing hypoxic composite neonatal morbidity was higher among LGA and SGA when compared to AGA infants, increased with maternal pre-pregnancy BMI (with the exception of underweight women) and when GWG exceeded IOM recommendations. Instead, the risk of traumatic composite neonatal morbidity increased with birthweight, among obese class 1 women, while it dropped when GWG remained below IOM recommendations.

**Table 4:** Risk of hypoxic and traumatic composite neonatal morbidity according to pre-pregnancy BMI, GWG and birthweight (n = 110594).

	Hypoxic composite neonatal morbidity		Traumatic composite neonatal morbidity	
	OR*	p	OR*	p
Birthweight category				
** **AGA	-		-	
** **LGA	1.46 [1.36–1.58]	<0.01	3.54 [3.18–3.94]	<0.01
** **SGA	1.48 [1.37–1.59]	<0.01	0.45 [0.36–0.57]	<0.01
BMI Category				
** **Normal weight	-		-	
** **Underweight	0.93 [0.83–1.04]	0.19	1.08 [0.86–1.36]	0.47
** **Overweight	1.26 [1.18–1.34]	<0.01	1.09 [0.97–1.23]	0.16
** **Obesity Class 1	1.34 [1.24–1.45]	<0.01	1.27 [1.10–1.48]	<0.01
** **Obesity Class 2	1.69 [1.52–1.88]	<0.01	1.12 [0.90–1.40]	0.31
** **Obesity Class 3	1.79 [1.58–2.02]	<0.01	0.92 [0.69–1.22]	0.56
GWG according to IOM recommendations				
** **Within	-		-	
** **Below	0.94 [0.87–1.01]	0.09	0.72 [0.62–0.85]	<0.01
** **Above	1.08 [1.02–1.14]	0.01	1.00 [0.90–1.12]	0.95

* OR was estimated using logistic regression adjusted for race, marital status, parity, smoke, alcohol use, and sex of the infant. Maternal education, insurance status and anemia were not included as they were missing in >10% of the women.

The multivariable analyses estimating at entry of care the risk of hypoxic and traumatic neonatal events are displayed in Table 5.

**Table 5:** Risk of hypoxic and traumatic composite neonatal morbidity at entry of care according to pre-pregnancy BMI and GWG (n = 110594).

	Hypoxic composite neonatal morbidity		Traumatic composite neonatal morbidity	
	OR*	p	OR*	p
BMI Category				
** **Normal weight	-		-	
** **Underweight	0.94 [0.84–1.05]	0.28	1 [0.8–1.25]	1.00
** **Overweight	1.27 [1.19–1.35]	<0.01	1.14 [1.01–1.28]	0.03
** **Obesity Class 1	1.36 [1.25–1.47]	<0.01	1.4 [1.21–1.62]	<0.01
** **Obesity Class 2	1.72 [1.54–1.91]	<0.01	1.31 [1.05–1.63]	0.02
** **Obesity Class 3	1.84 [1.63–2.08]	<0.01	1.16 [0.87–1.54]	0.32
GWG according to IOM recommendations				
** **Within	-		-	
** **Below	0.95 [0.88–1.02]	0.19	0.67 [0.57–0.78]	<0.01
** **Above	1.09 [1.03–1.15]	<0.01	1.17 [1.05–1.3]	0.01

* OR was estimated using logistic regression adjusted for race, marital status, parity, smoke, alcohol use, and sex of the infant. Maternal education, insurance status and anemia were not included as they were missing in >10% of the women. Infants’ birthweight was not included as it is not available at entry of care.

The risk of hypoxic composite neonatal morbidity increased with maternal pre-pregnancy BMI (with the exception of underweight women) and if possible GWG exceeded IOM recommendations. Similarly, the risk of traumatic composite neonatal morbidity increased with maternal pre-pregnancy BMI (with the exception of underweight and obese class 3 women), and with possible GWG. Both models showed poor calibration and discrimination for hypoxic (HL goodness of fit p = 0.008 and AUC = 0.60, 95% CI 0.59–0.61) and traumatic (HL goodness of fit p = 0.719 and AUC = 0.58, 95% CI 0.57–0.59) composite morbidities. Increasing pre-pregnancy BMI had a stronger association with hypoxic morbidity than GWG: the model designed to assess the association between BMI and infant morbidity (that included all the covariates but maternal GWG) had a significantly higher AUC than the model testing the association between GWG and infant morbidity (that included all the covariates but BMI) (0.596 95%CI 0.59–0.6 vs 0.584 95%CI 0.577–0.59; p < 0.01). Instead, data did not show that maternal BMI and GWG differently affected traumatic morbidity as the AUC of the 2 models did not differ (0.563 95%CI 0.55–0.576 vs 0.573 95%CI 0.56–0.586; p = 0.1).

In all multivariable analyses no interactions were found between maternal BMI class and GWG.

## Discussion

Using a prospectively collected cohort of more than 100,000 deliveries, we showed that hypoxic neonatal events were more frequent than traumatic complications, the former being more common among SGA and LGA than AGA infants, the latter increasing with birthweight. Previous studies showed that as pre-pregnancy BMI and GWG raised, the risk of having LGA infants increased, while the risk of SGA dropped [1,2]. Instead, we found that maternal pre-pregnancy BMI and GWG affect infant morbidity differently from birthweight: increasing maternal BMI and GWG were associated to higher risks of hypoxic as well as traumatic morbidity among SGA, LGA and AGA infants. Furthermore, we showed that when risks were estimated at entry of care, increasing pre-pregnancy BMI had a stronger association to hypoxic morbidity than GWG, while maternal BMI and GWG equally affected traumatic morbidity.

Pre-pregnancy BMI becomes a fundamental focus for preconceptional counseling and an important target for interventions to prevent neonatal complications in future pregnancies: reproductive age women should be encouraged to normalize their weight preconceptionally, as this seems to be at least as effective as avoiding excessive weight gain to prevent neonatal morbidity.

Our findings add direct evidence to the work of Aune D et al, who conducted a systematic review and meta-analysis of cohort studies, showing that even modest increases in maternal BMI are associated with higher risks of fetal death, stillbirth, and perinatal death; however, the authors did not address the concomitant role of GWG on adverse neonatal outcomes [21]. Furthermore, the results of our study confirm that infants born to women with BMI > 30 kg/m^2^ have proportionally higher risks of hypoxic complications as maternal obesity class increases [21]. Overweight and obese women are more frequently affected by preeclampsia [24], gestational diabetes [14,25], type 2 diabetes [26], hypertensive disorders [27] and congenital anomalies [28], that affect neonatal health. These women also present with altered lipid metabolism, increased inflammatory responses, and endothelial dysfunction, that mimic the abnormal vascular profile typical of preeclampsia [29], favoring placental thrombosis and subsequently stillbirth [29–31]. Finally, maternal obesity is also characterized by increased pre-pregnancy maternal insulin resistance and oxidative stress, that seem to affect early placental function [32].

Previous studies found a U-shaped association between adverse pregnancy outcomes and total GWG, indicating that GWG outside IOM recommendations may be detrimental [2,33]; instead, we found that GWG above (but not below) IOM recommendations is associated with hypoxic neonatal events, and that GWG correlates in a linear fashion with traumatic neonatal events. As opposed to previous works, our study outcomes did not include SGA, whose prevalence increases with insufficient weight gain [3,34]. However, in a recent retrospective US cohort of more than 15 million mother-infant dyads, Wang et al found that inadequate GWG was associated with increased risks of significant morbidity and mortality of the newborn infant [9]. Differently from our work, the authors did include pregnancies resulting in preterm deliveries, which are characterized by worse outcomes [35] and by smaller GWGs due to their shorter duration. Bodnar et al previously converted women’s total weight gain to gestational age-standardized z scores, which enabled the relationship between weight gain and infant death to be isolated from the association with preterm birth [11]. The authors built a multivariable logistic regression model for each pre-pregnancy BMI category (underweight, normal weight, overweight, obesity class 1, 2, and 3) to estimate the association between GWG and infant mortality, showing that both insufficient and excessive GWG led to higher risks of infant death. When compared to our work, the authors adopted a different primary outcome (infant mortality as opposed to hypoxic and traumatic events) and a different methodological approach.

Different lines of evidence suggest that offspring of inadequate GWG are at increased health risks due to prematurity, intrauterine growth restriction [3,33], and adverse intrauterine fetal programming [36]. However, the placenta is a plastic organ that counteracts exogenous insults. In women exposed to Dutch Famine during pregnancy, compensatory growth of the placenta induced by maternal caloric restriction maintained consistent fetal nutrition until parturition, so that birthweights were, up to a certain extent, normal [37]. It was estimated that to achieve the recommended healthy GWG, underweight, normal weight and overweight women need to store amounts of daily calories inversely related to their BMI, while women with obesity do not need to store energy at all, as they can mobilize calories from their adipose tissue [38]. Therefore, we can speculate that in industrialized countries, overfeeding and significant pregnancy weight gain pose a greater threat to fetal and neonatal wellbeing than limited weight gain or weight neutrality, as even with restricted caloric supply maternal metabolic pathways are designed to favor fetal over maternal growth. Several studies showed the benefit of limited GWG in obese women. A meta-analysis of almost 740,000 obese women suggested that a 1–5 Kg GWG for obese class 2 women and a 0 Kg GWG for obese class 3 women could improve fetal growth and caesarean delivery outcomes [39]. An observational study on 337,590 women reported that weight neutrality for obese class 1 women, up to 4 Kg weight loss for obese class 2, and up to 5 Kg weight loss for obese class 3 women were associated with a reduced risk of hypertension and emergency caesarean delivery [40]. The effect of restricted GWG on pregnancy outcomes was also tested in small prospective studies: none reported any harm, while a few indicated potential benefits; however their small sample size limited inference [41–43]. Finally, a population-based cohort study of 15760 pregnancies followed for a median of 7.9 years addressed how GWG affects the risk of a composite outcome including stillbirth, infant death, LGA, SGA, preterm birth, unplanned caesarean delivery, gestational diabetes, pre-eclampsia, excess postpartum weight retention, and maternal cardiometabolic disease after pregnancy. In pregnancies with class 1 or 2 obesity, GWG below the lower limit of the IOM recommendations as well as weight loss did not increase the risk of the adverse composite outcome. Instead, in pregnancies with class 3 obesity, below-than-recommended or negative weight gain decreased the risk of the composite outcome [44].

Current recommendations on GWG in pregnancy consider SGA and LGA infants as equivalent and aim at lowering their combined risk of occurrence [2,3,8]. Previous studies showed that SGA infants are more susceptible to hypoxic events [5,6], while LGA are at greater risk of traumatic incidents [5,7]. Our analyses indicated that SGA and LGA infants share similar risks of hypoxic adverse events, whereas the latter are at higher risk of traumatic complications; this is particularly relevant considering that heavier mothers-to-be and significant GWG are associated with worse perinatal and LGA risks. Although the management of pregnancies resulting in growth-restricted fetuses is well established [6] uncertainties remain when fetal growth is accelerated [7]. Our findings indicate the need of further studies on interventions, (i.e. planned timing and mode of delivery) that could prevent the higher morbidity we observed among LGA infants.

The use of a large obstetric cohort with detailed clinical data gave strength to our conclusions and allowed us to address differences in outcomes based on obesity severity. However, our research is not free of limitations. Some socio-demographic or pregnancy characteristics were not available for a significant proportion of the cohort, therefore their contribution could not be assessed in the multivariable analysis. Various types of morbidity were considered as equally important when merged into the composite outcomes, even if their implications for the health of the newborn are different. The few cases of the observed perinatal deaths prevented us from analyzing this specific outcome alone, while the relatively small number of underweight mother-to-be may affected the analyses on this category. Our conclusions only regard term pregnancies: as the information available on GWG was pregnancy specific we decided to exclude preterm deliveries because we could not determine if limited weight gain was an effect or a potential cause of shorter pregnancies. Finally, the poor discrimination ability of our multivariable models indicates that a significant proportion of infant complications can’t be estimated early in pregnancy based on socio-demographic, medical and obstetric characteristics, but may be due to events that occur later in pregnancy or at the time of delivery.

## Conclusions

This large retrospective cohort study found that hypoxic was more frequent than traumatic neonatal morbidity. We also showed that pre-pregnancy BMI, and to a less extent, GWG were associated with increased risks of hypoxic neonatal morbidity, while they equally affected traumatic events. Finally, LGA infants presented similar risks of hypoxic events when compared to SGA, but greater risks of traumatic complications. Given the obesity epidemic, women who plan pregnancies, who became pregnant, as well as obstetric providers, should take these findings into consideration to improve infants’ health. Our report and previous studies [21,25–28] suggested that higher maternal BMI is clearly associated with increased infant morbidity and mortality. As maternal BMI depends on both pre-pregnancy weight and GWG, women should aim to achieve a normal BMI while they plan future pregnancies, and should also avoid excessive weight gain once they become pregnant. As the evidence that increased risk of neonatal morbidity depends on high maternal BMI and excessive GWG become clearer, future studies should investigate the effect of low BMI and BMI-specific GWG on the occurrence of hypoxic and traumatic neonatal events. Furthermore, as maternal BMI and GWG explain only a portion of neonatal morbidities, further studies are necessary to identify other conditions that may affect their occurrence either antenatally or at the time of delivery.

## Supporting information

S1 Checklist (DOCX)

S1 Table STROBE checklist. (DOCX)
