# Supplementary material for: Do maternal BMI and gestational weight gain equally affect the risk of infant hypoxic and traumatic events?
Source: PLoS One. 2024 Aug 6;19(8):e0308441. doi: 10.1371/journal.pone.0308441 (PMC11302857; doi:10.1371/journal.pone.0308441)
Supplement: S1 Checklist — (DOCX) [file pone.0308441.s001.docx]

***PLOS ONE* Clinical Studies Checklist**

***PLOS ONE* manuscript number: _** PONE-D-24-05314**______________________**

| **Complete the following if your study involved human participants or human subjects’ data. These questions should be addressed for prospective and retrospective studies.** | | |
| --- | --- | --- |
| 1. | Did you obtain ethics approval for this study?   - If yes, please upload (file type “Other”) the original approval document you received from your ethics committee. If the original document is in another language, please also provide an English translation.   ___ Uploaded __X_ N/A   - If you did not obtain ethical approval, please explain why this was not required.  \| From the Methods section “We used data from the Consortium on Safe Labor, a multicenter retrospective observational study that abstracted detailed labor and delivery information from electronic medical records in 12 US clinical centers (with 19 hospitals), from 2002 to 2008, available from the National Institute of Child and Human Development Data and Specimen Hub (NICHD DASH, 17). The original study was approved by the institutional review boards (IRB) of all participating institutions; a detailed description of its design was provided elsewhere (18). As the survey represented a retrospective analysis of medical records, the IRBs of all participating institutions waived the requirement for informed consent; furthermore, data were fully anonymized before they were accessed for the primary analysis. As we conducted a secondary analysis of previously published fully anonymized data, the study protocol was exempted from IRB approval at our institution”. \| \| --- \| |  |
| 2. | If your study involved human participants, please report in the Methods section when participants were recruited to the study.  _X__ Completed ___ N/A |  |
| 3. | If you are reporting a study of medical records or archived samples, please report in the Methods section the date range in which human subjects’ data/samples were collected and the date(s) when you conducted this study.  __X_ Completed ___ N/A |  |
| 4. | Please specify in the Methods section whether authors had access to information that could identify individual participants during or after data collection.  _X__ Completed ___ N/A |  |
| 5. | If you are reporting an observational study – i.e. cohort, case-control, and cross-sectional studies – we recommend that the work is reported as per the requirements of the STROBE guidelines, and that you provide a completed STROBE checklist as a Supporting Information file with your submission.  The STROBE checklist was developed to improve the reporting of observational human subjects research, and is available here: <http://strobe-statement.org/fileadmin/Strobe/uploads/checklists/STROBE_checklist_v4_combined_PlosMedicine.docx>.  __X_ Completed ___ N/A |  |
| 6. | Please ensure that the author list and Corresponding Author entered in Editorial Manager match the author list and Corresponding Author in your manuscript file.  _X__ Completed |  |
